# Persistent impact of *in utero* nanoparticle exposure on metabolic and endocrine outcomes in adult rats fed a high-fat diet

**DOI:** 10.1016/j.reprotox.2025.109140

**Published:** 2025-12-13

**Authors:** Russell Hunter, Teresa Gluth, Kate Seman, Travis Goldsmith, Riley Nett, Victoria Nist, Allison Dunn, Eric Kelly, Elizabeth Bowdridge

**Affiliations:** West Virginia University, Department of Physiology, Pharmacology, and Toxicology, Morgantown, West Virginia, United States

**Keywords:** Nanoparticulate, Gestation, Development, Metabolism, Developmental origins of, health and disease, Inhalation, Sexual dimorphism

## Abstract

Gestational nano titanium-dioxide (nano-TiO_2_) exposure causes reduced fetal size and multi-generational reproductive effects in females. The current study utilized a whole-body nano-TiO_2_ inhalation exposure model in pregnant Sprague-Dawley rats coupled with a high fat diet (HFD) fed to adult offspring to examine lasting effects of *in-utero* exposures on weight gain, metabolic function, and endocrine perturbations. Sexually dimorphic responses in weight gain were observed whereby exposed HFD males gained less weight than their air HFD counterparts (435 g ± 9.8 vs. 505 g ± 14.5; p < 0.05), but there was no weight difference between air and exposed HFD females. Males and females presented with exposure driven decreases in glucose tolerance, such that HFD exposed animals were significantly more glucose intolerant(−6985AUC±1763). Hypothalamic gene expression of the melanocortin receptor 3 was significantly increased in nano-TiO_2_ males (1453 %) and significantly decreased in nano-TiO_2_ females (29.07 %) compared to grain-based diet (GBD) controls. Hepatic mitochondrial activity was affected in sexually dependent manner, with females exhibiting changes in Complexes I, II, III, and V, and males only showing differences in Complex I activity. Finally, exposed males had smaller testicular mass (3.685 mg ± 0.0895 in GBD; 3.358 mg±0.0786 in HFD; (3.480 mg± 0.2023 in GBD, 3.380 mg± 0.0777) and reduced testosterone (7.75 μg/nl ± 1.965 in GBD; 3.62 μg/nl±2.084 in HFD; (9.943 μg/nl±1.97 in GBD, 4.28 μg/nl± 1.6845) compared to air males. Altogether, these data reflect how nanoparticulate driven differences in growth and development at birth can alter weight gain and metabolic function later in life in the face of a dietary challenge.

## 1. Introduction

Gestation and fetal development represent a particularly vulnerable period of life for both mother and child. Perturbations of the tightly controlled cellular and molecular processes of implantation, placentation, and fetogenesis may result in severe or fatal maternal and fetal consequences. Serious complications such as low birthweight, preterm birth [1,2], intrauterine growth restriction (IUGR), preeclampsia, and perinatal mortality have toxicant-specific risks with air pollutant exposure [3–7], which include nano- and ultrafine particulate matter. Additionally, size at birth, which is heavily dependent upon the placenta’s ability to properly deliver nutrients, is a significant predictor of life expectancy [8]. The capability of the placenta to efficiently adapt morphology and functionality when faced with a toxicological insult has lasting adult health outcomes in the offspring.

Of particular concern are the potential adverse effects of nanoparticulate exposure during gestation. Following the industrial revolution, human exposure to nanosized particles (NPs, <100 nm) has increased dramatically. Environmental toxicants such as particulate matter from combustion in traffic and industry, as well as metals can cause direct damage to the placental unit or cross the placenta into the developing fetus. The propensity of a toxicant to traverse the placenta largely favors low molecular weight, however other physiological factors may influence translocation [9]. Due to the heightened size-specific translocation potential of NPs, risks to fetal health are of particular concern. Nanomaterials such as nanoparticulate titanium dioxide (nano-TiO_2_), gold NPs, and recently nanoplastics have been shown in murine models to pass through the placenta and accumulate in fetal tissue [10–14]. However, it should be noted that some NPs, such as cadmium quantum dots, can be blocked at the level of the placenta and still cause fetal malformations through indirect mechanisms such as cell apoptosis and oxidative stress [15].

As a vascular toxicant, pulmonary nano-TiO_2_ exposure decreases endothelium-dependent relaxation in the thoracic aorta and femoral arteries, and endothelium-independent relaxation impairment in the thoracic aorta [16]. Additionally, maternal nano-TiO_2_ inhalation exposure during gestation increases placental vascular resistance and impairs umbilical vascular reactivity [17]. Furthermore, *in utero* exposure to nano-TiO_2_ results in sexually dimorphic responses in fetoplacental outcomes. Exposed fetal females present with significantly smaller mass compared to their exposed male counterparts. Moreover, fetal females had significantly decreased junctional zone area and increased labyrinth zone area, indicating placental dysfunction [18]. Nano-TiO_2_ exposure also causes maternal redox dysregulation, leading to increased hepatic hydrogen peroxide (H_2_O_2_) and reduced glutathione. These changes in systemic redox signaling persisted into the F1 and F2 generations, illustrating multi-generational effects derived from maternal gestational nano-TiO_2_ exposure [19].

The exposure driven diminished fetal size raises questions of nutritional programming and developmental delays potentially stemming from central hypothalamic alterations due to *in utero* exposure. The hypothalamus is a critical region of the brain that maintains homeostasis through by regulating a variety of inputs including metabolism, behavior, and hormones. Although the blood brain barrier is intact in the hypothalamus, there are areas such as the median eminence and the arcuate nucleus (ARC), which are more permeable than other regions. Therefore, the ARC, which contains several neurons responsible for food intake and regulation, can directly interact with circulating hormones and other systemic signals or toxicants. The increased permeability of the ARC makes it a potential target and accumulation site for nanoparticles. Gold nanoparticles alter the feed intake response in murine *in vivo* models [20] and energy metabolism in *in vitro* models [21]. In humans, fetal growth trajectories and neurodevelopmental endpoints may be affected in babies that are small for their gestational age [22]. Amongst babies with IUGR, cognitive and motor functions are impaired [23], and 5 month old male F1 nano-TiO_2_ exposed rat offspring displayed significant impairments in working or short term memory and initial motivation [24]. Again, demonstrating that gestational nano-TiO_2_ exposure can cause central nervous system disruptions that span the lifetime of an individual.

Maternal inhalation exposure to nanoparticulate matter is a well-established risk factor implicated in vascular, reproductive, behavioral, metabolic and hepatic dysfunction in rodent offspring [18,19,24, 25]. Recently, the concept of initiating a second stressor to make any deficits become more pronounced when a second environmental factor interacts with the initial exposure have been explored. *In utero* exposure to nanoparticulate matter serves as a “first hit”, priming the developing fetus and increasing susceptibility to subsequent adverse exposure, or “second hit”, such as a high fat diet. Given that over 40 % of the United States population is considered obese, with almost 10 % presenting as severely obese [26], the introduction of a HFD postpubertaly after *in utero* exposure seems both timely and incredibly relevant to typical human exposure circumstances. Therefore, we hypothesize that postnatal stressors, such as dietary challenges during adulthood, may synergize with the prenatal priming event of gestational maternal exposure to exacerbate metabolic abnormalities. To test this hypothesis, the current study seeks to determine the potential interaction between gestational nano-TiO_2_ exposure *in utero* and how an additional insult in adulthood, specifically HFD, affects growth and metabolic function in male and female offspring.

## 2. Methods

### 2.1. Animal model

Female, Sprague–Dawley (SD) rats were purchased from Hilltop Laboratories (Scottdale, PA), and housed in an Assessment and Accreditation of Laboratory Animal Care (AAALAC) approved facility at West Virginia University (WVU) under a regulated temperature and 12:12 h light–dark cycle. Rats were randomly assigned to either filtered air (n = 6) or nano-TiO_2_ (n = 6) exposure groups and acclimated for 48–72-h before mating. Rats had ad libitum access to food and water throughout the acclimation period. Vaginal smears were used to confirm estrus prior to pairing, and pregnancy was confirmed via presence of a seminal plug. Weights of pregnant dams were recorded weekly. Pups were housed with dams for 21 days postnatally and weaned based on sex. Weight-matched pups from typical litters were selected for subsequent analysis. Litters with less than five pups were omitted as these are considered atypical and may alter metabolic status of the pups [27].

### 2.2. Diets

At 12 weeks of age, animals were randomly assigned to receive either a grain-based diet (GBD, 18 % fat, T2918, Teklad INC^®^), or high fat diet (HFD, 60 % fat, D12492, Research Diets INC^®^; Supplemental Table 1). A total of 32 (n = 8 per group) animals were selected and divided evenly into 4 filtered air groups (FA GBD Male, FA GBD Female, FA HFD Male, FA HFD Female), while 48 animals (n = 12 per group) were divided evenly amongst the nano-TiO_2_ exposed group (nano-TiO_2_ GBD Male, nano-TiO_2_ GBD Female, nano-TiO_2_ HFD Male, nano-TiO_2_ HFD Female). Complete formulations including micronutrient values can be found in Supplemental Table 1. The HFD was refreshed every 2–3 days to limit oxidation and prevent spoiling. Animals were weighed weekly for 12 weeks; at which time final weights were taken and animals were euthanized. All procedures were approved by the Institutional Animal Care and Use Committee of West Virginia University.

### 2.3. Inhalation exposure

Nano-TiO_2_ aerosols were generated using a high-pressure acoustical generator (HPAG, IEStechno, Morgantown, WV). The output of the generator was fed into a Venturi pump (JS-60 M, Vaccon, Medway, MA) which further de-agglomerated the particles. The nano- TiO_2_ aerosol/air mix then entered the whole-body exposure chamber. A personal DataRAM (pDR-1500; Thermo Environmental Instruments Inc., Franklin, MA) was utilized to sample the exposure chamber air to determine the aerosol mass concentration in real-time. Feedback loops within the software automatically adjusted the acoustic energy to maintain a stable mass concentration during the exposure. Gravimetric measurements were conducted on Teflon filters concurrently with the DataRAM measurements to obtain a calibration factor. The gravimetric measurements were also conducted during each exposure to calculate the mass concentration measurements reported in the study. Bedding material soaked with water was used in the exposure chamber to maintain humidity (30–70 %) during exposures. Filtered air animals were exposed to HEPA filtered air only (25 mL/min) with similar temperature and humidity chamber conditions. Air and nano-TiO_2_ exposures were conducted simultaneously in the same room, with the only difference between exposures being the presence of the nano-TiO_2_ nanoparticles.

Inhalation exposures in F0 dams lasted for 6 non-consecutive days after GD 10 to decrease animal stress and possible pregnancy loss. The pregnant rats were exposed to an average target concentration of 12 mg/m^3^. While trace amounts of TiO_2_ may be ingested with grooming following exposures, there was no direct exposure of TiO_2_ to the offspring after birth for the duration of the study. This concentration of 12 mg/m^3^ was chosen to match our previous late gestation inhalation exposure studies [16,17] with a similar exposure paradigm (12 mg/m^3^, 6 h/exposure, 6 days) and with the last exposure conducted 24 h prior to sacrifice and experimentation. The particle size distributions of the aerosol were measured with multiple devices including: 1) scanning mobility particle sizer (SMPS 3938, TSI Inc., St. Paul, Minnesota), 2) aerodynamic particle sizer (APS 3321, TSI Inc., St. Paul, Minnesota), 3) electrical low-pressure Impactor (ELPI+, Dekati, Tampere, Finland), and 4) Nano Micro-Orifice Uniform Deposit Impactor (MOUDI 115 R, MSP Corp, Shoreview, MN). Log normal fits of the size distribution were conducted to calculate the count median diameter (CMD), mass median aerodynamic diameter (MMAD) and geometric standard deviation (GSD) of the distributions. MPPD Software (v 2.11, Arlington, VA) was used to estimate [28] the alveolar lung dose from the nano-TiO_2_ exposures based upon the aerosol characteristics and the breathing parameters of the rats.

### 2.4. Glucose tolerance test

At 20 weeks of age, rats were fasted for 6 h prior to glucose tolerance testing (GTT). Animals had fasting glucose measured via handheld glucose monitoring system (Contour^®^) before an intraperitoneal injection of filter-sterilized 2 g/kg glucose in 0.9 % NaCl, similar to previous studies [29]. After which, samples were taken every 20 min for 2 h.

### 2.5. Plasma and tissue collection

At 24 weeks of age, all animals were deeply anesthetized via isoflurane prior to euthanasia via cardiac exsanguination. At euthanasia, blood samples were taken from each animal and collected in a K2 EDTA blood tube (BD 367856) for centrifugation and plasma collection. Tissues were immediately excised, rinsed in ice-cold PBS, weighed and snap frozen for future analysis.

### 2.6. Testosterone ELISA

Plasma was assayed for testosterone concentration using a commercially available mouse/rat testosterone ELISA (Calbiotech; TE187S-100) according to manufacturer protocol.

### 2.7. Xanthine oxidase activity assay

Xanthine oxidase (XO) was measured in plasma as previously described [28]. Briefly, plasma samples (10 μL) were diluted 30X in 300 μL of phosphate buffered saline. Potential urate oxidase (uricase) activity was inhibited by the addition of oxonic acid (100 μM) to ensure UA was not metabolized. Total UA production in 60 min at 37 °C in the presence of xanthine (75 μM) served as the basis for quantification of XO activity. Allopurinol (100 μM) was used in parallel samples to inhibit XO to establish base-line UA levels. Following incubation, protein was precipitated with ice cold acetonitrile. The samples were centrifuged for 12 min at 13,200 x g, at 4 °C. Following centrifugation, the supernatant was removed, placed in borosilicate glass tubes, dried (60 min), resuspended in isocratic mobile phase (300 μL), and filtered through 0.20 μm nylon membrane filter unit into 11 mm plastic snap top auto-sample vials. The UA content was measured by electrochemical detection (Vanquish UltiMate 3000 ECD-3000RS) coupled to reverse-phase HPLC using a C18 column (150 ×4.6 mm, 3 μm particle size, Luna Phenomenex) and isocratic mobile phase (50 mM sodium dihydrogen phosphate, 4 mM dodecyltrimethylammonium chloride, 2.5 % methanol, pH 7.0). One unit of activity (U) was defined as 1 μmole/min urate formed at 37°C and pH 7.4.

### 2.8. Real-time PCR

Hypothalamic tissue was collected immediately following euthanasia, flash frozen in liquid nitrogen, and stored at −80°C until processed for mRNA extraction. Total RNA was extracted via the RNeasy Kit (Qiagen, Hilden, Germany) based on manufacturer’s recommendations. Total RNA was then transcribed to cDNA via the High-Capacity RNA-to-cDNA kit (Thermo Fisher Scientific).

Using real-time PCR, genes involved in diet and appetite control were assessed. Specifically, Melanocortin Receptors 3 and 4 (*Mc3r, Mc4r*) [22,23], Pro-opiomelanocortin (*Pomc*) [24], Agouti-related neuropeptide(*Agrp*) [30], NeuropeptideY (*Npy*) [31], Gamma-aminobutyric Acid (*Gaba*) [32], Leptin (*Lep*) [33], Ghrelin (*Ghrl*) [34], Leptin Receptor (*Lepr*) [35], and nuclear receptor subfamily 5 group A member 1 (*Nr5a1*) [36]. Genes were tested against ribosomal subunit protein 18 s(*Rn18s*) [37]. Primers were purchased from Integrated DNA Technologies (Coralville, Iowa); primer sequences, and references are listed in Table 1. Samples were analyzed in triplicate using iTAQ Universal SYBR Green Supermix (Bio-Rad Laboratories, Hercules, California). Relative fold changes in expression of candidate genes were obtained using the 2 −ΔΔ*C*_t_ method [38]. *C*_t_ values were used to calculate Δ*C*_t_ values for genes of interest [*C*_t_(test) – *C*_t_(housekeeping)]. The housekeeping gene for normalization was Ribosomal subunit 18S (*Rn18s*). Statistical representation for each gene is based on fold change with respect to Rn18s.

### 2.9. Mitochondrial function assays

Electron transport chain (ETC) Complex activities (I, II, III, IV, and V) were measured in liver and reproductive tissue of adult offspring as previously described [39]. Liver and reproductive tissue was homogenized using the Polytron PowerGen 500 S1 tissue homogenizer (Fisher Scientific, Hampton, NH) in NP-40 buffer (20 mM Tris, 137 mM NaCl, 10 % Glycerol, 1 % Triton × 100, 2 mM EDTA) [26]. Samples were centrifuged for 10 min at 10 000 xg (4 °C) and the supernatant was used for the activity assays. The protein homogenates were used to measure activities of ETC Complexes I, II, III, IV, and V (ATP synthase). ETC Complex I and III activities were determined by measuring the reduction of decylubiquinone (I) and cytochrome c (III) in liver and reproductive tissue protein lysate of adult offspring. ETC Complex II activity was measured using the reduction of dichlorophenolindophenol. ETC Complex IV activity was determined by measuring the oxidation of reduced cytochrome c, while Complex V activity was determined by measuring oligomycin-sensitive ATPase activity through pyruvate kinase and phosphoenolpyruvate in liver and reproductive tissue protein lysate of adult offspring. The Molecular Devices Flex Station 3 Multi-Mode microplate reader (Sunnyvale, CA) was used to measure all assays spectrophotometrically. Protein content was normalized using the Bradford method, with bovine serum albumin protein assay standards. Final values were expressed as Unit/nanogram (I-IV) or milligram (V) of protein, where Unit = nanomoles of substrate oxidized (minute −1).

### 2.10. Statistical analysis

Weight gain was analyzed via two-way analysis of variance (ANOVA) with repeated measures. All other physiological characteristics were assessed by two-way mixed-effects ANOVA. If statistical significance was achieved, a Tukey post hoc test was utilized. All data are reported as mean ± SEM, unless otherwise stated. Significance was set at p ≤ 0.05. All data was analyzed in GraphPad Prism 10.

## 3. Results

### 3.1. Characterization of titanium dioxide nanoparticulate aerosol

**Fig. 1.**
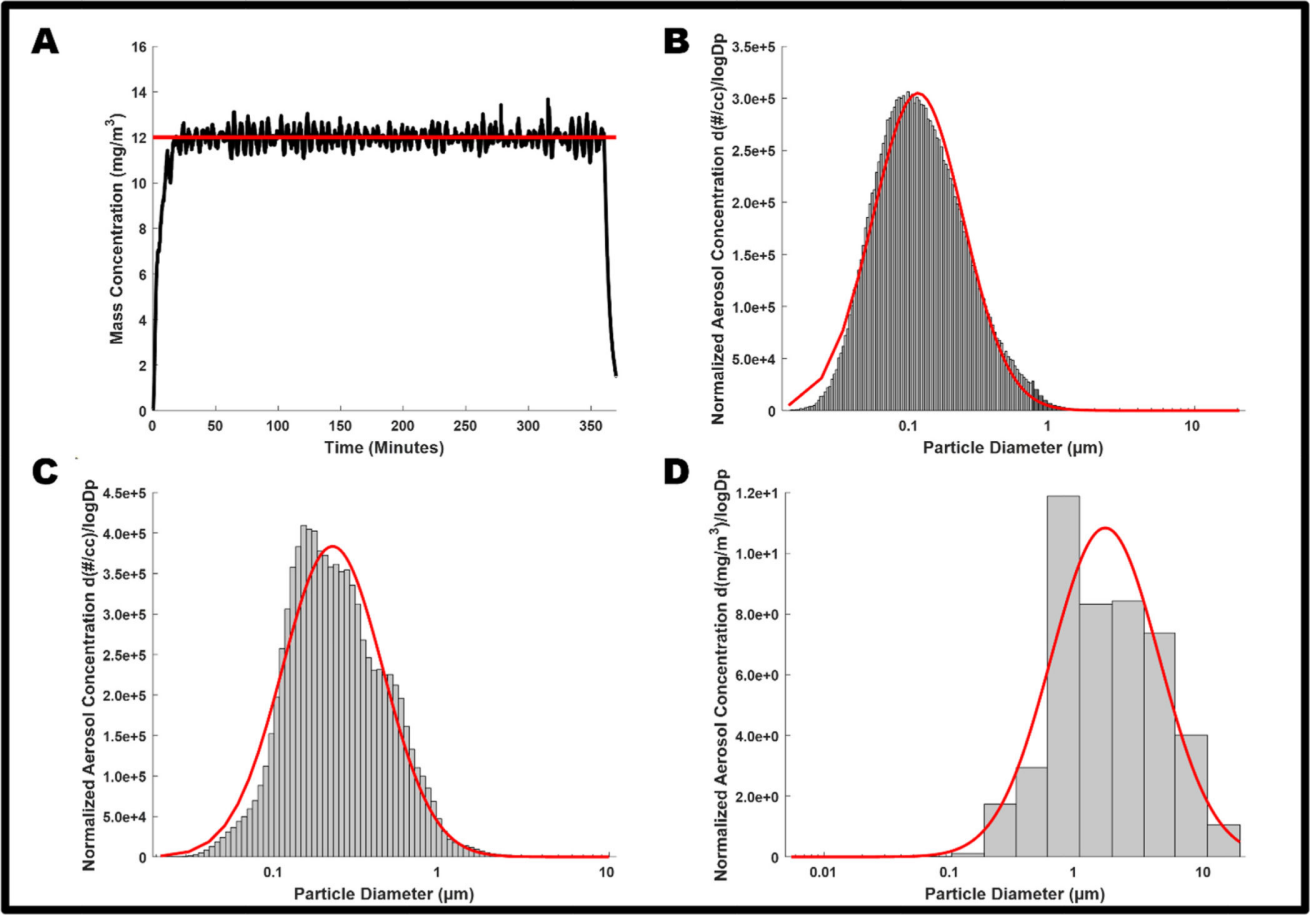


Average real-time aerosol mass concentration over the course of exposures was 12.05 mg/m^3^ with a standard deviation of 1.26 (Fig. 1A). The combined SMPS and APS measurements indicated a CMD of 115 nm with a GSD of 2.07 (Fig. 1B). ELPI assessment of the aerosol aerodynamic diameter showed a CMD of 224 nm with a GSD of 2.00 (Fig. 1C). The MOUDI measurement yielded a MMAD of 1.59 μm and a GSD of 2.65 (Fig. 1D). The size and distribution of these particulates are similar to previous characterizations for this exposure paradigm [18, 40]. The morphology of the nano-TiO_2_ agglomerates has been previously characterized extensively with electron microscopy [17]. MPPD deposition modeling, based on the aerosol and animal breathing characteristics, indicated a daily alveolar deposition of 31.26 μg with a corresponding 6-day deposition of 187.53 μg.

### 3.2. Sexually dimorphic responses in weight gain for in-utero nano-TiO_2_ exposed animals

Maternal exposure to nano-TiO_2_ has previously been reported to cause reduced fetal weight [19], with early life weight gain in postnatal pups showing a similar reduction [41]. Weight gain of the animals in the current study was reported in an earlier publication examining cardiovascular development [39]. A summary of those results is provided herein (Table 2). To avoid confounding normal development and puberty onset, all F1 animals were maintained on the GBD until 12 weeks of age. Both males and females exposed to nano-TiO_2_ *in-utero* showed significantly reduced body weight compared to sham-exposed controls at 6 weeks of age (p < 0.05). Males and females gained significantly more weight on HFD when compared to matched GBD counterparts, however males on HFD born to nano-TiO_2_ exposed mothers gained significantly less compared to maternally air-exposed animals on HFD (p < 0.05).

### 3.3. Increases in glucose tolerance impairment from animals exposed to in-utero nano-TiO_2_ and high fat diet

**Fig. 2.**
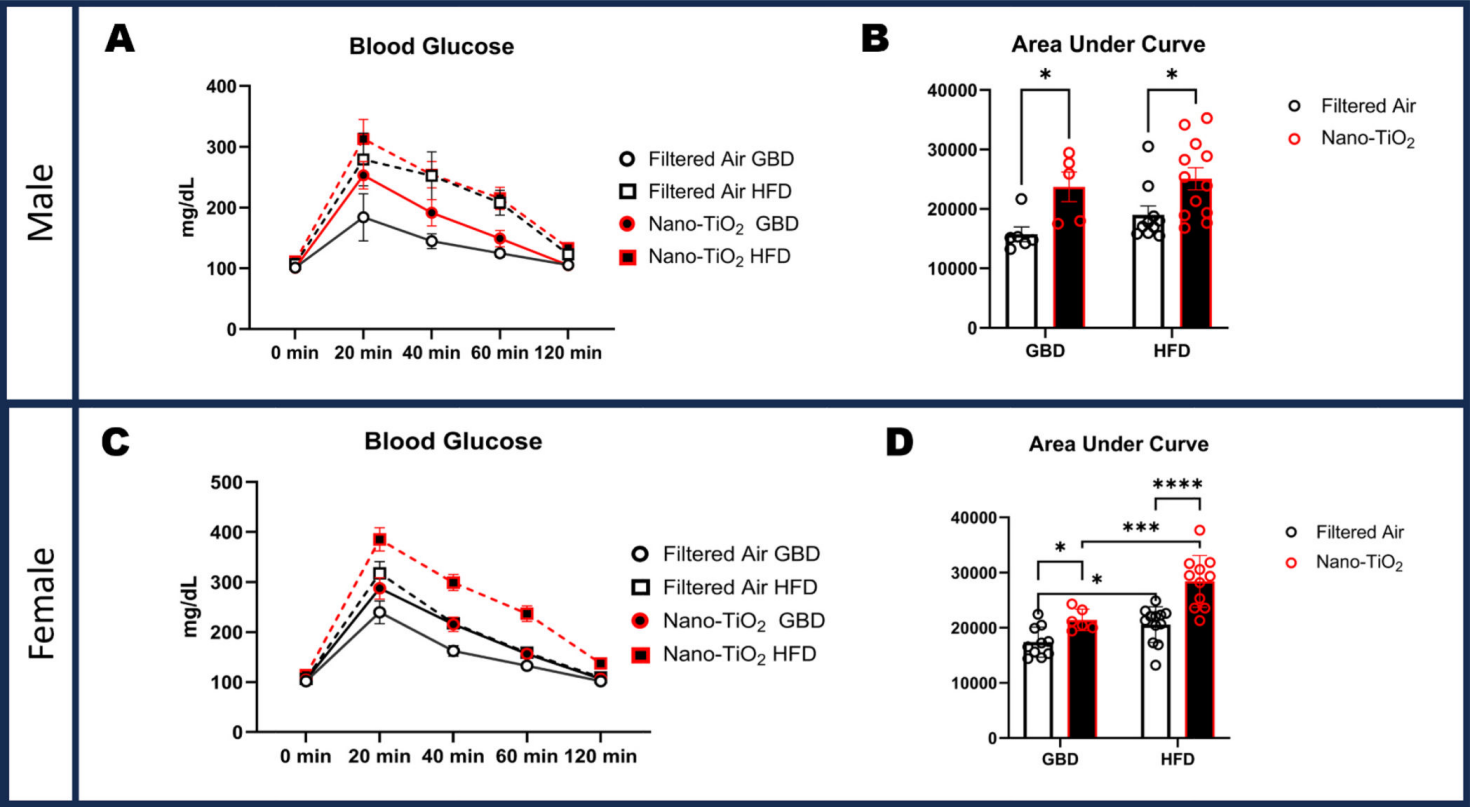


At 20 weeks of age and 8 weeks of respective diets, animals were assessed for glucose tolerance. Males showed significant exposure-driven impairment in glucose tolerance in the GBD group (23714 ± 2502 AUC) and HFD group (25079 ± 1858AUC) when compared to nano-TiO_2_ exposed groups (15741 ± 1229AUC, and 19020 ±1484AUC, respectively; Fig. 2A-B). Females showed a similar pattern in significant exposure driven impairments, with the GBD group (17411 ± 860AUC) and HFD group (20572 ± 935AUC) for the filtered air exposure having significantly greater glucose tolerance compared to their nano-TiO_2_ exposed counterparts (21430 ± 791AUC, and 28415 ± 1420AUC, respectively). The two-hit interaction produced a profound difference in the females, with the nano-TiO_2_ HFD (28415 ± 1420.1251) being significantly more glucose intolerant in the nano-TiO_2_ GBD group (21430AUC± 791.1468; Fig. 2C-D).

### 3.4. Differential organ weights resulting from diet and in-utero exposure

**Fig. 3.**
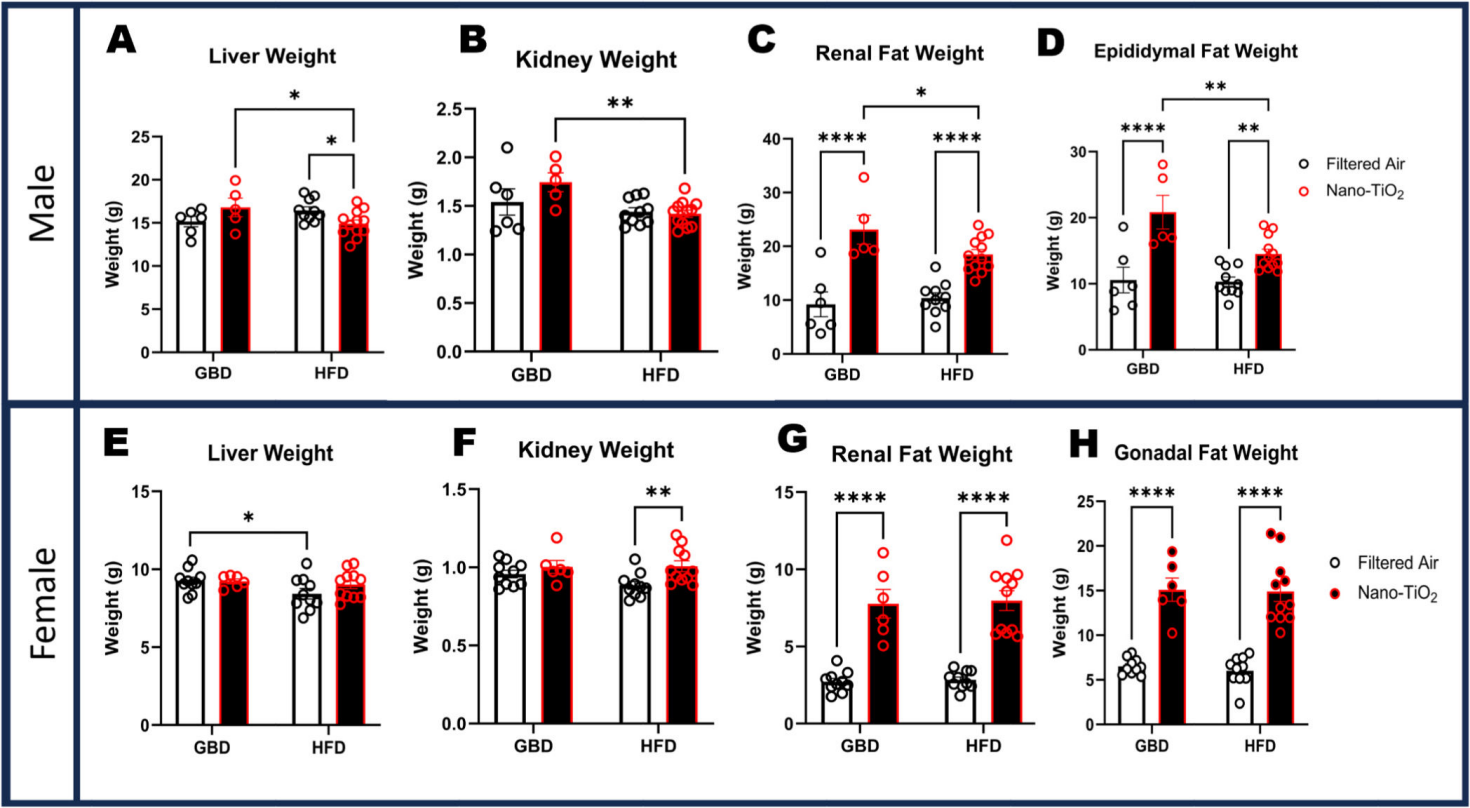


Following euthanasia via cardiac exsanguination, organs were immediately rinsed in ice-cold PBS and weighed. Heart weights in males (Supplemental Figure 3A-B), were significantly higher for both HFD groups in terms of unweighted mass. Lung weight showed a similar significant increase for males in the nano-TiO_2_ exposed group, while females showed the greatest increase in lung index in the air-HFD group (Supplemental Figure 3D). Male liver weights were significantly reduced in the nano-TiO_2_ exposed HFD group, and females had a significantly lower liver weight by diet in the filtered air group (Fig. 3A&E). Kidney weight was significantly decreased in the HFD exposed group compared to filtered air, which was in contrast to female kidney weight that was increased amongst the exposed HFD group compared to air-HFD (Fig. 3B&F). Unweighted renal fat weight and indexed to bodyweight were significant higher in exposed groups for both sex and dietary comparisons (Fig. 3C&G). Epididymal and gonadal fat was significantly elevated in exposed groups for both sexes and diets (Fig. 3D&H).

### 3.5. Changes in gene expression of appetite regulatory genes in hypothalamus

**Fig. 4.**
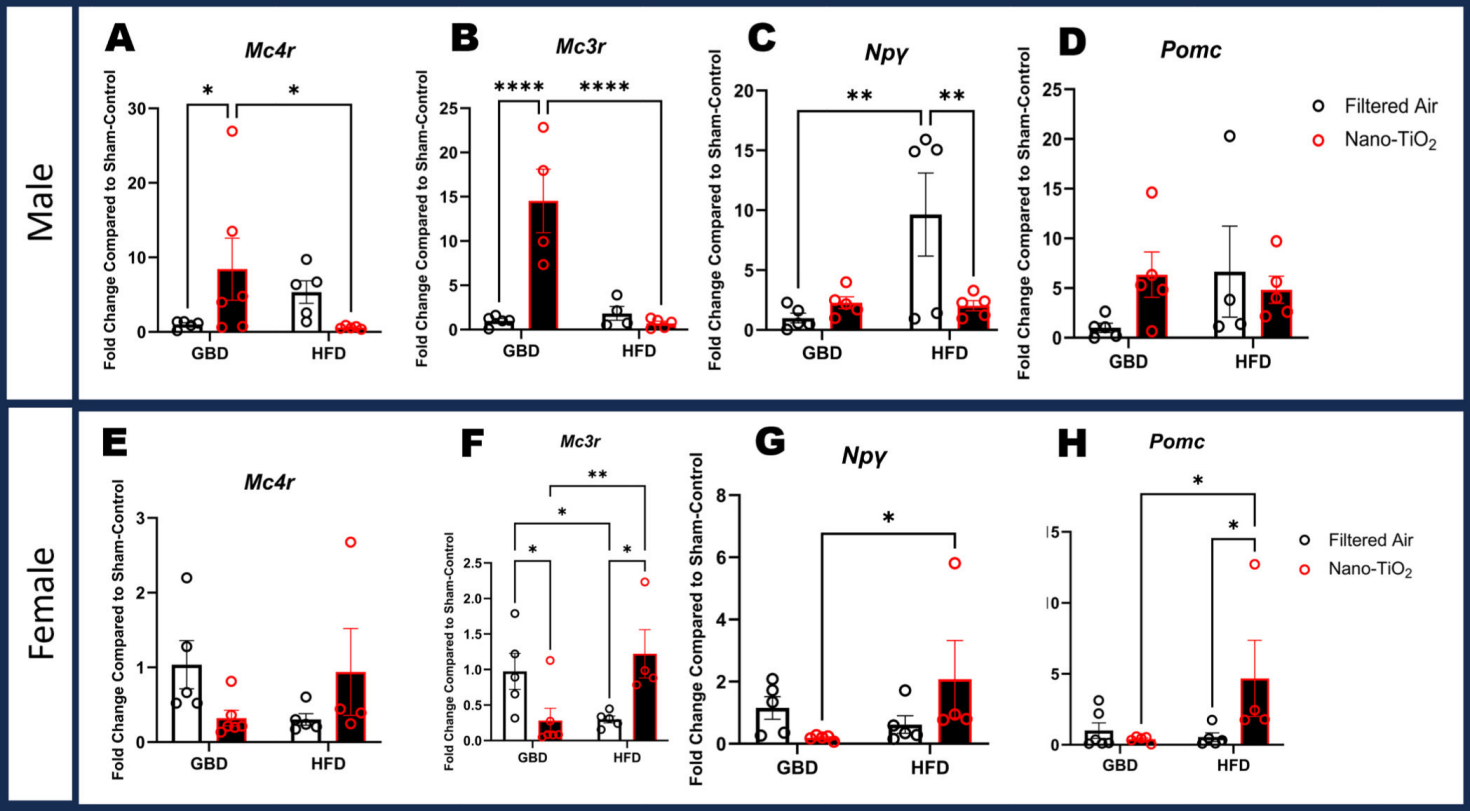


Several hypothalamic appetite genes were examined to determine what role they may have played in the differential weight gain between sexes. *Mc4r* expression among males was significantly increased in the nano-TiO_2_ GBD group compared to the filtered air group. *Mc4r* expression in the HFD males was lower in the nano-TiO_2_ compared to filtered air (Fig. 4A). No significant differences for *Mc4r* expression were observed in females (Fig. 4E). Males exhibited a similar pattern in *Mc3r* expression, with a significant increase in the nano-TiO_2_ exposed GBD group compared to GBD filtered air and HFD nano-TiO_2_, and no difference between filtered air and nano-TiO_2_ HFD groups. (Fig. 4B). In females, *Mc3r* expression was significantly decreased in the GBD group by exposure and significantly increased in the HFD group by exposure resulting in an exposure by diet interaction (Fig. 4F). *Npγ* expression in filtered air males was significantly increased in the HFD compared to GBD. HFD nano-TiO_2_ expression was also significantly decreased compared to the HFD filtered air (Fig. 4C). Females showed a significant diet-induced increase in *Npγ* expression, with HFD nano-TiO_2_ being increased compared to GBD nano-TiO_2_ (Fig. 4G). No significant differences in *Pomc* levels were observed in males (Fig. 4D). Female *Pomc* expression in the HFD nano-TiO_2_ group was significantly increased when compared to both GBD nanoTiO_2_ as well as HFD filtered air (Fig. 4H).

### 3.6. Hepatic mitochondrial function and circulating redox factors differentially affects females following in-utero nano-TiO_2_ exposure

**Fig. 5.**
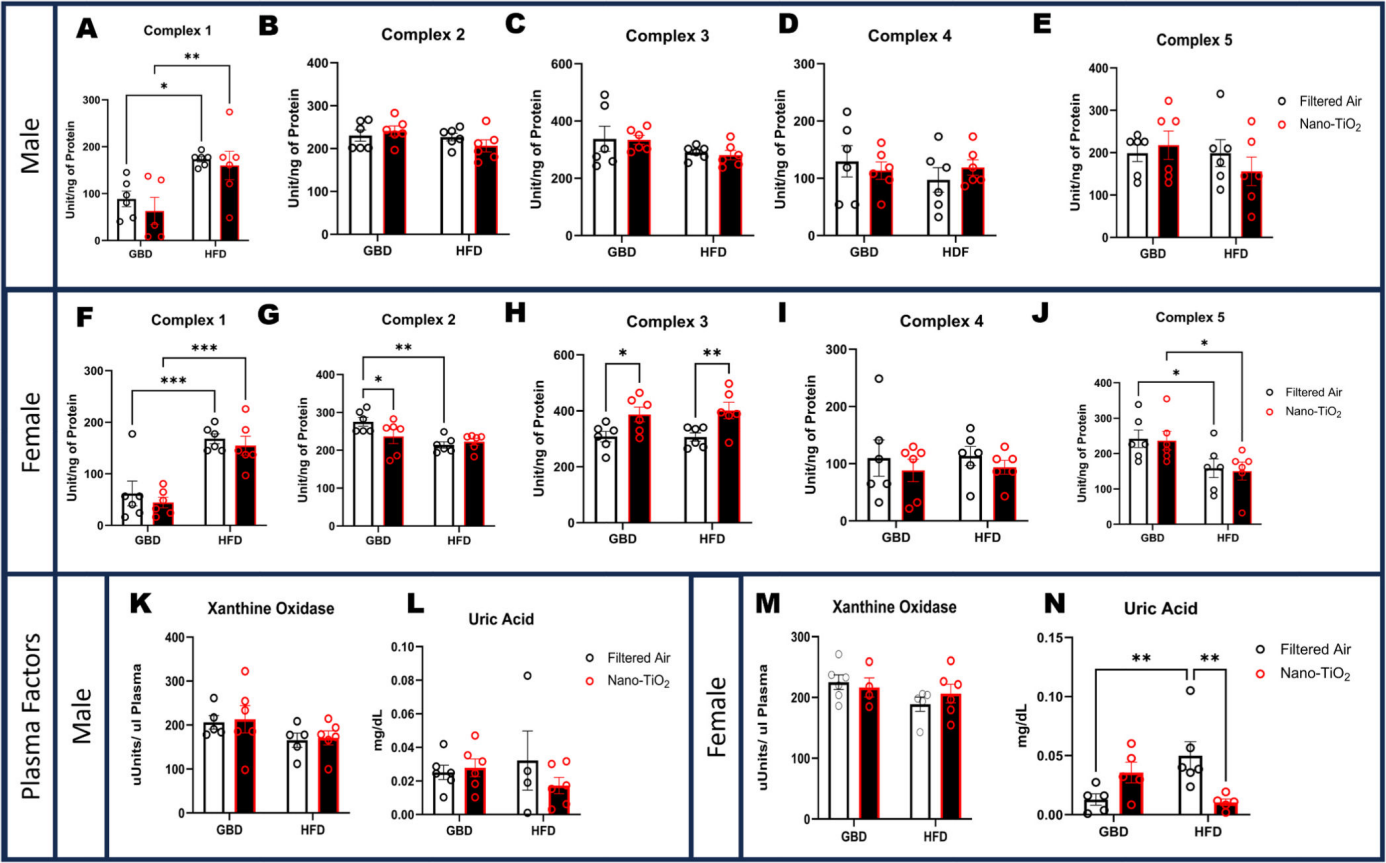


At euthanasia, sections of the right lobe of the liver were excised and frozen for mitochondrial function analysis. In males, Complex I activity was significantly increased in HFD groups, with an exposure interaction whereby nano-TiO_2_ has increased activity compared to the GBD group (Fig. 5A). There were no significant differences in activity in the remaining mitochondrial complexes in male livers (Fig. 5B-E). In females, significant differences in hepatic mitochondrial function were detected in Complexes I, II, III, and V, but not in Complex IV (Fig. 5I). Female hepatic mitochondrial Complex I activity was significantly increased in HFD groups similarly to the males (Fig. 5F). Female Complex II activity presented with significantly decreased activity in the nano-TiO_2_ exposed GBD group and HFD filtered air compared to the GBD filtered air group (Fig. 5G). A significant exposure driven change was observed in Complex III, where both nano-TiO_2_ exposed groups had higher activity compared to filtered air within their respective dietary groups. (Fig. 5H). Complex V activity was significantly decreased in both HFD groups compared to GBD (Fig. 5J). Plasma levels of xanthine oxidase (XO) activity and uric acid (UA) were assayed from plasma samples taken at euthanasia. Males did not exhibit significant differences in XO activity or UA levels across dietary or exposure conditions (Fig. 5K-L). Similarly, there were no significant differences in XO activity in females (Fig. 5M). However, female plasma UA levels were significantly increased in filtered air on the HFD compared to GBD. Nano-TiO_2_ HFD animals presented with significantly decreased UA levels compared to filtered air HFD counterparts (Fig. 5N).

### 3.7. Testis size and testosterone production are reduced following in-utero nano-TiO_2_ exposure

**Fig. 6.**
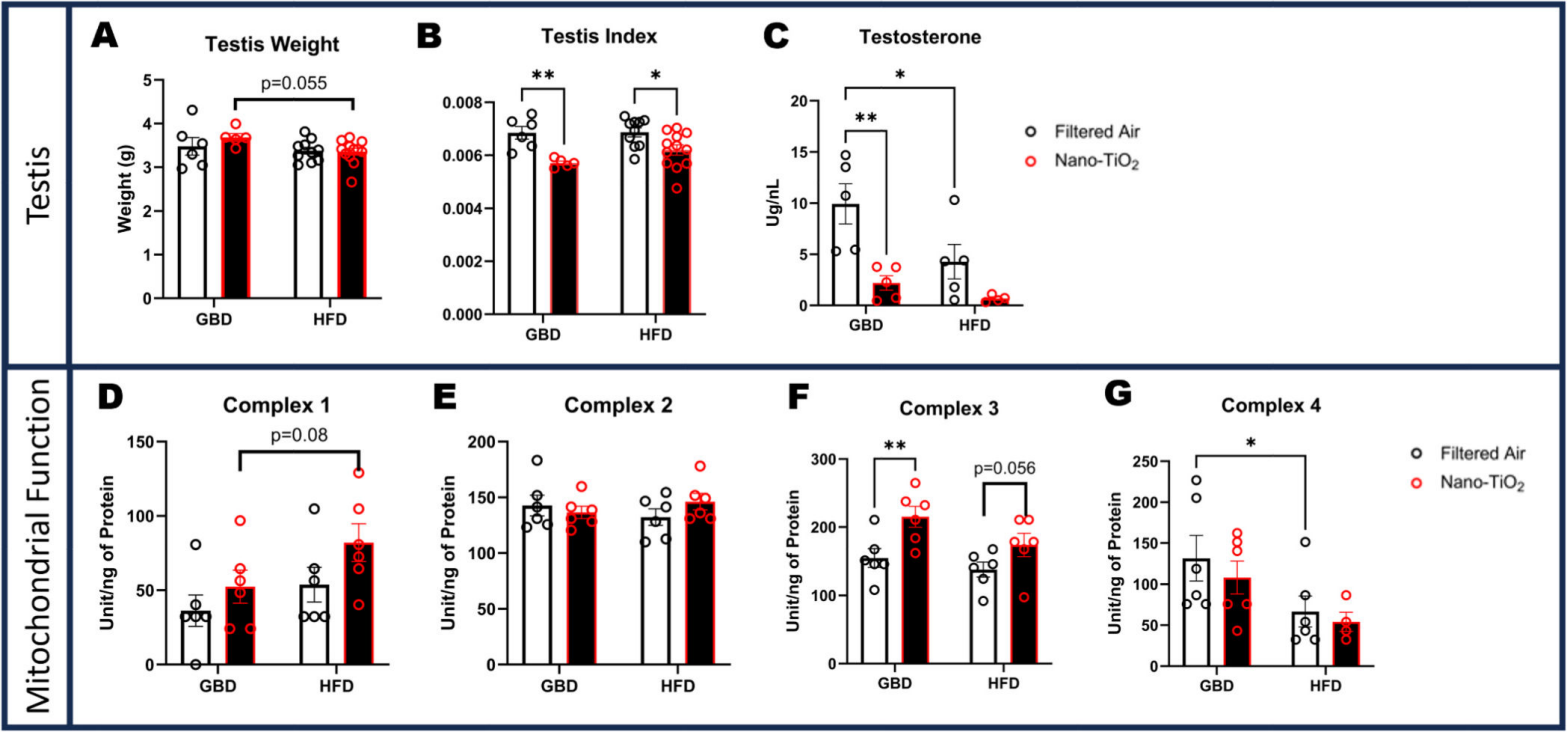


Testis weight at euthanasia revealed a trending decrease in testis size in the nano-TiO_2_ exposed group when compared to filtered air (Fig. 6A). When indexed to body weight both GBD and HFD groups had significant decreases in testis size in the nano-TiO_2_ groups compared to their filtered air counterparts (Fig. 6B). Plasma testosterone at euthanasia was significantly decreased for both GBD nano-TiO_2_ and HFD filtered air groups when compared to the GBD filtered air group (Fig. 6C). There were no significant differences in mitochondrial activity in the testis between groups, however there were trends for increased activity in Complex I and III in the exposed HFD males compared to filtered air HFD counterparts (Fig. 6D-G).

### 3.8. Ovarian mitochondrial energetics are altered following in-utero nano-TiO_2_ exposure

**Fig. 7.**
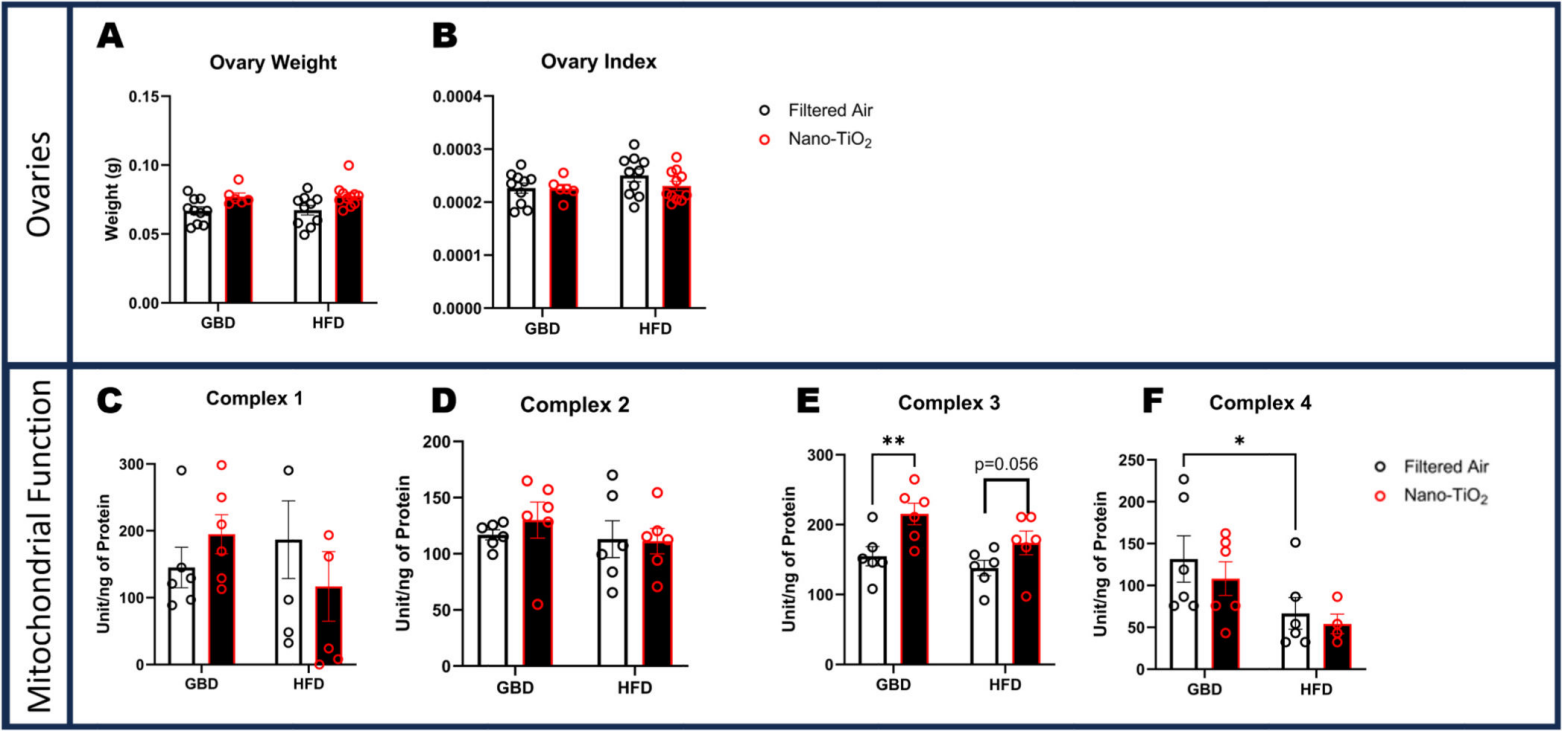


Ovarian weights were taken at time of euthanasia and showed no significant differences between groups by raw or indexed comparison (Fig. 7A-B). Ovarian mitochondrial activity was not significantly different between groups for Complexes I and II (Fig. 7C-D). However, Complex III activity was significantly increased in nano-TiO_2_ exposed GBD group. There was a trending increase with nano-TiO_2_ exposure for Complex III activity within the HFD group as well (Fig. 7E). Complex IV activity was significantly reduced in filtered air HFD compared to filtered air GBD (Fig. 7F).

## 4. Discussion

The two-hit hypothesis model employed in this study examines the complex interplay between *in-utero* exposure as a prenatal programming event, and dietary responsiveness in offspring as a result. As reported previously [41], birth weight for animals born to mothers exposed to nano-TiO_2_ is significantly reduced. Upon introduction of a HFD at 12 weeks postnatally, *in utero* exposed males exhibited reduced weight gain whereas females showed increased weight gain compared to air-exposed controls. Gestational exposure resulted in increased renal fat mass for both sexes, and epididymal, and gonadal fat masses respectively. Titanium nanoparticles, via intraperitoneal injections, have previously been shown to infiltrate into fat tissue and induce inflammatory signals [42]. At present, it remains unknown if deposition of particles from the gestational exposure are capable of accounting for these increased in fat pad masses at 24 weeks of age. The characterization of gestational exposure risk, and clinical guidance for early growth and development may be essential in combatting the increasing prevalence of obesity-related, metabolic and other noncommunicable diseases.

Gestational nano-TiO_2_ exposure produced significant changes to hepatic function and glucose metabolism in adult offspring [19]. Herein, both males and females presented with exposure-driven increases in glucose intolerance, with exposed HFD females showing the greatest glucose intolerance. Additionally, there was an exposure-driven increase in mitochondrial Complex III activity within the liver. Previously, we have shown that directly exposed dams and their F1 progeny at 8 weeks of age presented with upregulated H_2_O_2_ production capacity and diminished glutathione antioxidant defense in the liver [19]. Taken together, this illustrates the persistent hepatic and metabolic effects nanoparticulate exposure has across generations. Continued elucidation of mechanisms of maladaptive developmental programming due to prenatal nanoparticulate insult are needed to understand how gestational exposures lead to long-term adult health deficits in offspring.

Several differences in appetite regulatory genes in the hypothalamus were noted in this study. Our data shows sex-dependent differences in hypothalamic expression of *Mc3r* and *Mc4r*. Previous reports have demonstrated sex-dependent differences in *Mc4r* loss-of-function rats [43], and *Mc3r* in mice [44]. Additionally, intraperitoneal injections of nano-TiO_2_ caused a loss of appetite in rats [45], however, few studies have examined the interplay between nanoparticle inhalation exposure and appetite. The decrease in *Mc3r* and *Mc4r* expression in HFD exposed males may, at least in part, explain the lack of weight gain in this group when compared to filtered air HFD males. Additionally, prenatal nano-TiO_2_ exposure results in neurobehavioral deficits in locomotor activity, learning, and memory [46], which can culminate in increased depressive-like behaviors in adulthood [47]. The current investigation did not monitor behavior, locomotion, or metabolism, which may provide context to physiological or epigenetic drivers related to these differences in appetite and previously mentioned weight gain. Given the weight differences observed prior to dietary introduction, the role of appetite-modulating factors may provide useful insights for human growth and development for children born underweight. Future studies examining behavioral patterns and neural appetite programming at weaning may prove useful for uncovering adult food intake changes.

Males exposed to nano-TiO_2_ *in utero* had reduced testicular size relative to body weight and decreased testosterone production following exposure. Mitochondrial assessment of testicular tissue revealed a dietary driven response amongst the exposed males, where Complexes I and III had increased activity in the testes, similar to other studies of prediabetic Wistar rats [48]. High-energy is required for spermatogenesis, where increased Complex I activity may be required to maintain aerobic metabolism [49]. Previous studies have shown multigenerational effects of nano-TiO_2_ exposure on reproductive outcomes in females, such as litter size and circulating estrogen concentrations [19]. The current study did not find changes in ovarian size but did see increased Complex III activity in exposed females that was further increased by HFD. Given the hormonal and mitochondrial perturbations seen in the males, future studies evaluating sperm quality, quantity, morphology, and male libido should be undertaken.

Herein, we observed higher weight gain trajectories for males that increased further with the introduction of a HFD. Most likely these changes are due to increased energy intake, as well as reduced locomotor activity in males [50]. Nano-TiO_2_ exposure *in utero* reduced HFD-induced weight gain in males, whereas there was no effect of exposure in females. These effects correspond to the impaired glucose tolerance in exposed males, which leads to prolonged glucose in circulation that is converted to fat, and therefore increased fat and body mass. Nano-TiO_2_ exposure resulted in increased glucose intolerance regardless of sex or diet, however exposed HFD females exhibited increased glucose intolerance compared to sham exposed HFD females, which is of interest considering there was no significant change in body mass. There was however an increase in fat mass deposition in the renal and gonadal fat pads of these exposed females. Female livers presented with more robust mitochondrial differences than males, including exposure driven increases in Complex III activity, and HFD induced reductions in Complex V activity. Complex V activity decline disrupts ATP synthesis, compromising hepatocellular function, and further increasing energy demand [51]. Altogether, these perturbations in metabolic markers indicate a pronounced sexual dimorphism in an adult HFD challenge after *in utero* exposure to particulate matter.

In summary, data presented herein illustrate clear sex-dependent differences in weight gain and hepatic mitochondrial activity following *in-utero* nano-TiO_2_ exposure. Females presented with more robust interactions between diet and exposure, showing altered hepatic metabolism through mitochondrial function and glucose intolerance. More work is needed to define the role of prenatal nanoparticulate exposure on metabolic, endocrine, and hypothalamic development that results in lifelong consequences in the offspring. Future studies focused on epigenetic mechanisms, pre-pubertal dietary challenge introduction, and behavioral changes will help unravel the myriad of adult health deficits observed due to *in utero* particulate exposure.

## Supplementary Material

1

2

## Figures and Tables

**Table 1:** Primer sequences for rtPCR experiments.

Primer	Forward Primer (5′−3′)	Reverse Primer (3′−5′)	Length (bp)	References

MC3R	CCA CGT TCA TGC CAT TGC AAC C	CGT GAT GTT CAT CGT CTA CTC	1098	26
MC4R	ATG AAC TCC ACC CAC CAC	CAT AGC ATC CTC CGT CTG	1888	27
POMC	TCC GAG AAG AGC CAG ACG	GCC TTG GAG TGA GAA GAC CC	1180	28
AgRP	GCA AGG ATC AAC AAG CAA	GAA CAG GGC CTG GTC AGA	6103	29
NPY	GCT TGA AGA CCC TTC CAT GTG GTG	GGC GGA GTC CAG CCT AGT GG	567	30
GABA	CAC GAA GAA GGA GGA GAA G	CAG ATG GCA AGA GTC AGG	4658	31
LEP	GTC TGG TCC ATC TTG GAC AAA CTC AGA ATG	CAT TCT GAG TTT GTC CAA GAT GGA CCA GAC	3298	32
GHRL	GTC ATC TGT CCT CAC CAC CAA	GTA GAT GTG GGG GCT TAG GG	6326	33
LepR	CAA CAA GCA TGG GCT GCA GTG ACA TTA GAG	CTC TAA TGT CAC TGC AGC CCA TGC TTG TTG	7675	34
NR5A1	AGA CAA GTG CAC CCC ATT TC	ACC ATC ACC AAC CGC TAA AC	3066	35
Rn18s	GTC CCC CAA CTT CTT AGA G	CAC CTA CGG AAA CCT TGT TAC	1872	36

**Table 2:** Weight gain statistics for animals on study (summarized from Hunter et al., 2025). Baseline weights (top) and final weights (middle) analyzed by 2-Way ANOVA, reported as Mean (Standard Error). Cumulative Weight gain (bottom) reported as repeated measures ANOVA.

6 Weeks		Filtered Air	Nano-TiO_2_	P Value	Summary			Filtered Air	Nano-TiO_2_	P Value	Summary
	Male	137 (3.272)	106.4 (1.482)	< 0.0001	****		Female	116.1 (1.839)	94 (2.962)	< 0.0001	****

	Male	Filtered Air	Nano-TiO_2_	P Value	Summary		Female	Filtered Air	Nano-TiO_2_	P Value	Summary
24 Weeks	GBD	506.7 (16.06)	492.6 (7.978)	0.7462	ns		GBD	294.8 (3.238)	268.0 (3.590)	0.1306	ns
	HFD	645.0 (18.97)	544.2 (10.18)	< 0.0001	****		HFD	342.3 (7.269)	338.2 (8.182)	0.9903	ns
	P Value	< 0.0001	0.0002				P Value	0.0063	< 0.0001		
	Summary	****	***				Summary	**	****		
	Male	Filtered Air	Nano-TiO_2_	P Value	Summary		Female	Filtered Air	Nano-TiO_2_	P Value	Summary
Cumulative Weight Gain	GBD	371.5 (12.53)	389.1 (7.393)	0.6372	ns		GBD	180.8 (2.988)	178.6 (4.870)	0.9799	ns
	HFD	505.8 (16.45)	435.3 (9.805)	0.031	*		HFD	222.7 (4.856)	240.5 (5.033)	0.0948	ns
	P Value	0.0009	0.0064				P Value	0.0002	< 0.0001		
	Summary	***	**				Summary	***	****		

## Data Availability

Data will be made available on request.

## Appendix A. Supporting information

Supplementary data associated with this article can be found in the online version at doi:10.1016/j.reprotox.2025.109140.
