# Supplementary material for: Persistent impact of in utero nanoparticle exposure on metabolic and endocrine outcomes in adult rats fed a high-fat diet
Source: Reprod Toxicol. Author manuscript; Available in PMC 2026 Mar 4. (PMC12959621; doi:10.1016/j.reprotox.2025.109140)
Supplement: 2 [file NIHMS2132187-supplement-2.docx]

|  | **Grain Based Diet** | **High Fat Diet** |
| --- | --- | --- |
| Protein | 18.4 | 26.2 |
| Carbohydrate | 44.2 | 25.6 |
| Fiber | 3.8 | 6.5 |
| Fat | 6 | 34.9 |
| Mineral | 5.5 | 6.5 |
| Vitamin | 3.5 | 0.4 |

**Supplemental Table 1.** Comparison of nutritional components of diets used in studies. Vales expressed as percent of total contribution to formula by weight.
